# The Possible Role of Probiotic Supplementation in Inflammation: A Narrative Review

**DOI:** 10.3390/microorganisms11092160

**Published:** 2023-08-26

**Authors:** Alessandro Colletti, Marzia Pellizzato, Arrigo Francesco Cicero

**Affiliations:** 1 Department of Science and Drug Technology, University of Turin, 10124 Turin, Italy; 2 Italian Society of Nutraceutical Formulators (SIFNut), 31033 Treviso, Italy; 3 Medical and Surgical Sciences Department, University of Bologna, 40126 Bologna, Italy; arrigo.cicero@unibo.it; 4 IRCCS AOUBO, 40138 Bologna, Italy

**Keywords:** probiotics, inflammation, aging, oxidative stress, urinary tract infections, cardiovascular disease, gut microbiota

## Abstract

The fine balance between symbiotic and potentially opportunistic and/or pathogenic microorganisms can undergo quantitative alterations, which, when associated with low intestinal biodiversity, could be responsible for the development of gut inflammation and the so-called “intestinal dysbiosis”. This condition is characterized by the disbalance of a fine synergistic mechanism involving the mucosal barrier, the intestinal neuroendocrine system, and the immune system that results in an acute inflammatory response induced by different causes, including viral or bacterial infections of the digestive tract. More frequently, however, dysbiosis is induced slowly and subtly by subliminal causal factors, resulting in a chronic condition related to different diseases affecting the digestive tract and other organs and apparatuses. Studies on animal models, together with studies on humans, highlight the significant role of the gut microbiota and microbiome in the occurrence of inflammatory conditions such as metabolic syndrome and cardiovascular diseases (CVDs); neurodegenerative, urologic, skin, liver, and kidney pathologies; and premature aging. The blood translocation of bacterial fragments has been found to be one of the processes linked to gut dysbiosis and responsible for the possible occurrence of “metabolic endotoxemia” and systemic inflammation, associated with an increased risk of oxidative stress and related diseases. In this context, supplementation with different probiotic strains has been shown to restore gut eubiosis, especially if administered in long-term treatments. The aim of this review is to describe the anti-inflammatory effects of specific probiotic strains observed in clinical trials and the respective indications, highlighting the differences in efficacy depending on strain, formulation, time and duration of treatment, and dosage used.

## 1. Introduction

For many years, the digestive system was considered to only include the set of organs involved in the transformation of food to be absorbed and the elimination of waste metabolites derived from digestive processes [1]. Studies in recent decades, however, have shown that this system is much more complex, and it carries out many functions that have important systemic repercussions [2]. The intestinal ecosystem consists essentially of the intestinal mucosal barrier, intestinal immune system, intestinal neuroendocrine system (also called the “second brain”), and intestinal microbiota. The first three elements constitute the “intestinal barrier”, which is a truly bidirectional selective filter. The enterocytes and the goblet cells, with a double layer of mucus, constitute the mucosal barrier, which represents the largest interface of the organism, together with the respiratory system, having a surface of 300–400 m^2^ in an adult subject [3]. The mucosal barrier of the gut separates the contents of the intestinal lumen (microbiota and occasional germs, food residues, secretions of the various digestive districts, and xenobiotics) from the specific gut-associated lymphoid tissue (GALT) and the vascular pathway [4]. In recent decades, researchers have highlighted the close interaction of the intestinal nervous system, the immune system, enterocytes, and microorganisms that constitute the intestinal microbiota. Data published to date indicate that the bacteria, viruses, phages, fungi, and yeasts that comprise the intestinal microbiota contribute to the functionality of the bidirectional contact between the components of the brain–intestine axis, intervening in the communication between the second brain and the main one [5,6]. In addition, studies have drawn attention to other bidirectional communications with other extra-intestinal organs, such as in the case of the intestine–skin [7], intestine–liver [8], intestine–kidney [9], intestine–heart [10], and intestine–bladder axes [11]. In this context, a healthy gut microbiota has an immense antioxidative and anti-inflammatory role, while an altered gut microbiota is associated with increasing oxidative stress and inflammation, well known to be correlated with several chronic diseases [12].

### 1.1. The Intestinal Microbiota

The intestinal microbiota is an ecosystem characterized by a set of ecological niches of different microbial populations, consisting of bacteria, phages, fungi, yeasts, and viruses. In recent decades, the bacterial component has been studied, albeit with difficulty because it includes thousands of species and hundreds of thousands of genes. It is believed that the number of species identifiable in the human intestinal microbiota is more than 1000 [13,14], with a genome comprising 600,000 to 3.3 million genes [15]. The microbiota of each individual contains >1000 species, and it is believed that there is a core of 57 species common to the subspecies *Homo sapiens sapiens* [16].

The four main phyla that compose the intestinal microbiota are *Firmicutes*, *Bacteroidetes*, *Actinobacteria*, and *Proteobacteria* [17]. *Firmicutes* and *Bacteroidetes* comprise about 90% of the colon’s bacterial population [18], while the remaining species, although always present, represent only 1–5% [19].

The composition of gut microbiota is generally subjected to small temporary variations depending on the lifestyle and physiological or pathological conditions of the subject, the consumption of medications, and diet. However, when both qualitative and quantitative microbiota balances are deeply modified, this results in a condition called “intestinal dysbiosis” [20].

### 1.2. Intestinal Eubiosis

Intestinal eubiosis is the condition in which the numerous and complex microbial communities that colonize the digestive system are in equilibrium, contributing to the state of health of the organism through metabolic and enzymatic activities that compensate for functions that the host is unable to perform or that can only perform insufficiently [21]. The functions performed by the gut microbiota in the eubiosis state include the degradation of nutrients introduced by the diet, the production of amino acids and essential vitamins, the maintenance of metabolic balance and energy homeostasis, the correct development and functioning of the immune system, the protection of anatomic and functional integrity of the intestinal wall, the degradation of xenobiotics, contribution to the maintenance of cognitive efficiency through the production of molecules that influence brain activity, and the promotion of hormonal balance through the synthesis of molecules with organotropism toward the endocrine organs [22].

However, the state of eubiosis may vary in a para-physiological way in relation to some factors related to the host and the environment in which they live. The most relevant factors are age [23], diet (especially the quantity and type of fiber introduced), and genotype [24]. In particular, the biodiversity of intestinal microbiota is strongly conditioned by the diet both in humans and other mammals. It is believed that dietary variations are at the base of approximately 60% of structural variations in the gut microbiota compared with approximately 12% that are due to genetic factors [25,26].

The microbiota has a kaleidoscopic composition that changes from individual to individual and, to a much smaller extent, in the same individual depending on age, comorbidities, lifestyle, and dietary variations. The balance of the different biological niches that coexist in this symbiotic relation guarantees the defense, metabolic, and structural functions of the microbiota [27].

### 1.3. Intestinal Dysbiosis

The term intestinal dysbiosis indicates a generic qualitative alteration but is also a quantitative indication of the different genera, species, and strains of the bacteria present in the intestine of an individual. This alteration causes inflammation and oxidative stress, in addition to the loss of balance between symbiotic and potentially pathogenic microorganisms with serious perturbation of the protective function of the mucosal barrier, the structural function of tight junctions, and all metabolic and enzymatic activities performed by the microbiota [28,29]. Dysbiosis can occur acutely as in the case of bacterial or viral infections of the digestive system or acute diarrhea related to the administration of drugs such as antibiotics [30]. More often, however, the causes that indicate this phenomenon arise more subtly, slowly configuring a chronic course of dysbiosis; they are, as is more and more frequently demonstrated in the literature, correlated to many diseases of the digestive system and were considered unreliable organs and apparatuses until a few years ago [31,32]. In this context, gut dysbiosis is characterized by the increase in different inflammatory mediators that may initiate pathological processes that can lead to microbiota aging and different chronic disorders [33]. Although it is difficult to determine a single causal pathway of systemic inflammation, this condition, associated with oxidative stress, is known to be highly expressed in subjects with intestinal dysbiosis and advanced age [34].

In addition to the abovementioned factors, the incongruous diet, food sensitivity, infections and infestations, the assumption of specific drugs (antibiotics, immunosuppressants, chemotherapeutic agents, proton pump inhibitors, and long-acting corticoids), and alterations in the immune response can exacerbate the state of dysbiosis.

**Figure 1:**
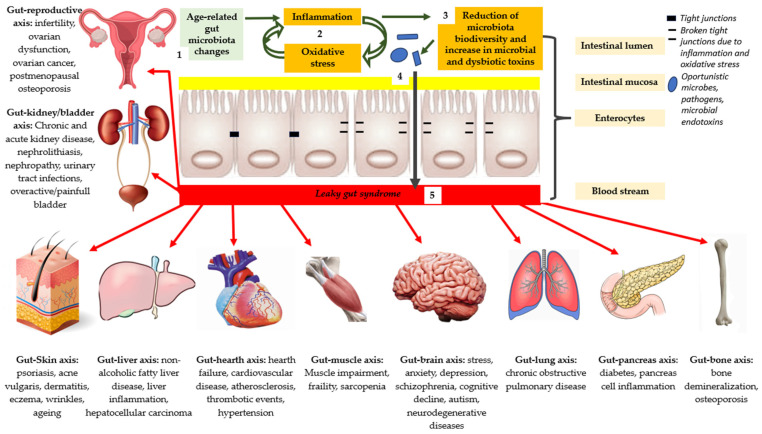
Aspects that characterize the syndrome of impaired intestinal permeability.

Chronic dysbiosis is defined as the chronic low-grade inflammation of the mucosal wall, leading to the deterioration of the tight junction selective function with the activation and overload of the immune system and, finally, the passage of harmful molecules of allergens and microorganisms, and toxicity in the circulatory torrent, which is then also spread to other organs. This clinical picture constitutes what is currently referred to as the “leaky gut syndrome” or syndrome of impaired intestinal permeability (Figure 1) [35].

It has been shown that the increase in permeability and the loss of selectivity of the mucosal barrier result in the emergence of pathologies loaded with other systems or even systemic diseases (Table 1) [36,37,38].

### 1.4. Role of Ox-Inflammaging on Intestinal Dysbiosis

The elderly population is growing year by year; in 2020, for the first time, people aged 60 and older outnumbered children aged 5 and younger [39]. The main manifestation of aging is low-grade chronic inflammation (LGCI), also known as inflammaging [40].

Epidemiological studies have indicated an increase in plasma levels of proinflammatory cytokines such as interleukin-6 (IL-6) and tumor necrosis factor-α (TNF-α) in the elderly population compared with young people [41]. The LGCI is associated with an increased risk of chronic diseases, disability, and mortality in elderly subjects [42]. On the basis of this condition, several pathogenetic mechanisms have been proposed, including cell senescence, the dysregulation of innate immunity, and changes in gut microbiota integrity [43]. In this regard, enterocytes represent the first barrier against pathogens and opportunistic microorganisms, secreting different antimicrobial molecules such as mucins and defensins, in addition to their ability to communicate with immune cells [44]. Increasing evidence suggests that the age-related deterioration of the intestinal barrier against bacteria may contribute to inflammaging and age-related chronic health conditions [45]. Thus, it is known that important perturbations affect the gut microbiota during aging. In particular, the reduction in microbial biodiversity and intestinal integrity contributes to intestinal dysbiosis and its associated inflammation [46]. In this context, the reduction in the integrity of the intestinal epithelium in old age could exacerbate the increase in inflammatory triggers due to the leakage of gut bacteria and/or endotoxins in the systemic circulation [47]. There is consequently a close correlation between intestinal aging and inflammation, although the primum movens of inflammaging and whether it is related to gut barrier aging or microbiota changes remain unclear. Thus, an understanding of the effects of multiple deregulations in the intestinal microbiota in mediating inflammaging with advancing age is still insufficient.

Studies conducted in humans showed that dysbiosis leads to the translocation of microorganisms into the circulation system of aged individuals, predisposing these subjects to several inflammatory diseases [48]. The mechanisms underlying age-related inflammation were investigated in mice, transferring the aged microbiota from old to young mice. The results highlighted that the young mice with aged microbiota showed an exaggerated systemic inflammatory response, expressed as an increase in TNF-α (known for its role in alterations in gut epithelial permeability), monocyte chemoattractant protein (MCP)-1, interferon (IFN)-γ, and reactive oxygen species (ROS) and a reduction in short-chain fatty acids (which play a crucial anti-inflammatory role in blocking the activation of transcription factor NF-kB), causing the aggravation of inflammaging [49,50,51]. In addition, Kim and colleagues demonstrated a correlation between aging and gut microbiota lipopolysaccharide (LPS)-induced inflammation, suggesting the relationship between aging and intestinal dysbiosis and inflammation [52]. Moreover, in the aging population, there is a reduction in anti-inflammatory eubiotic microorganisms, including *Bifidobacterium* spp. and *Faecalibacterium prausnitzii*, which maintain immune tolerance in the gut, and an increase in proinflammatory microbes, such as *Streptococcus* spp., *Enterococcus* spp., *Staphylococcus* spp., and *Enterobacter* spp. [53]. In recent years, the analysis of the intestinal microbiota of ultra-centenarians demonstrated a significant upregulation of the proinflammatory cytokines (IL-6 and IL-8), which correlated with changes in gut microbiota composition (enrichment in *Proteobacteria* and a decrease in *Ruminococcus lactaris*), confirming that the age-related changes in the composition of microbiota could be relevant in age-related inflammaging [54].

## 2. Materials and Methods

A systematic search strategy was conducted for this narrative review, in order to identify trials in both the Cochrane Register of Controlled Trials (The Cochrane Collaboration, Oxford, UK) and MEDLINE (National Library of Medicine, Bethesda, Maryland, MD, USA), from January 1970 to June 2023. The terms ‘probiotics’, ‘inflammation’, ‘oxidative stress’, ‘microbiome’, ‘clinical trial’, and ‘human’ were included in the electronic search strategy.

Only articles written in English were eligible for inclusion in this review. Study protocols and abstracts of conferences were excluded.

After a general introduction with an overview of the role of gut microbiota, intestinal dysbiosis, and related inflammation and oxidative stress, specific sections for each anti-inflammatory and antioxidant indication provide a short description of the mechanism of action, the clinically observed effects, and the most relevant tolerability notes. The authors of the writing and reviewing panels filled in Declaration of Interest forms to provide any real or potential sources of conflicts of interest.

## 3. Results

This section describes the evidence of results obtained from the supplementation of specific probiotic strains against oxidative stress and inflammation of the cardiovascular system, the digestive apparatus, and the urinary tract.

### 3.1. Probiotics and Gut Inflammation and Oxidative Stress

**Figure 2:**
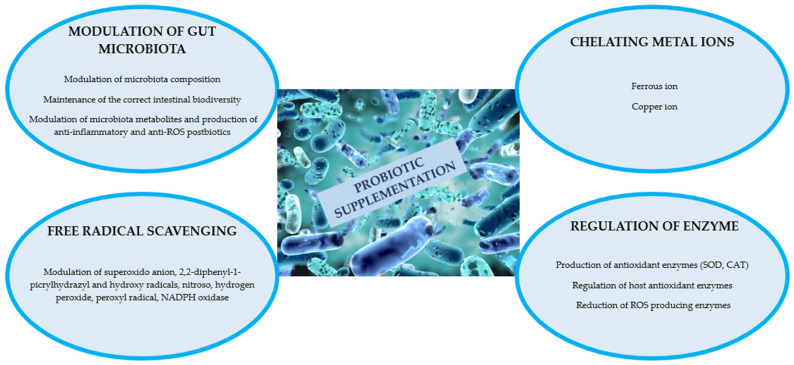
The proposed antiaging mechanisms of action of probiotics. ROS = reactive oxygen species, SOD = superoxide dismutase, CAT = catalase.

As illustrated in the Introduction section, several studies have highlighted the link between healthy aging and intestinal eubiosis. It has been reported that the microbiota composition of long-lived subjects is considerably different from that of young people and the frail elderly [55]. Probiotics are an appealing and effective approach to controlling various infectious diseases [56,57]. In this regard, specific probiotic strains in association with their fermented metabolites may be considered valid candidates for downregulating oxidative stress, which induces the aging process [58]. Probiotics have strong antioxidant properties and powerful redox systems, reducing the accumulation of ROS, which are a significant contributor to several disorders, such as inflammatory, cardiovascular, cerebrovascular, and degenerative diseases, as well as aging and cancer [59]. Several studies have shown that probiotics, such as *Lactobacillus* and *Bifidobacterium* spp., possess excellent antioxidant capacity to provide a certain degree of protection against oxidative stress, improving the balance in the oxidative and antioxidant systems and thus reducing the formation of free radicals by improving the antioxidant enzyme capacities [60]. Although the specific antioxidant mechanisms of action are not completely understood, eubiotic bacteria could show antioxidant activity through different mechanisms, including chelating metals, neutralizing ROS, increasing antioxidant enzyme levels, and modulating the microbiota (Figure 2) [61]. Several *lactobacillus* species exhibit high scavenging action against DPPH, O_2_^−^, and H_2_O_2_ in vitro; furthermore, they exert a nonenzymatic oxidative stress defense mechanism depending on the chelation of both Fe^2+^ and Cu^2+^, which represent the most active ions generated by ROS. Moreover, combined *Lactobacillus* strains showed that this supplementation may decrease NADPH oxidase (NOX) activity and *NOX-1* and *NOX-4* mRNA expression, which are major sources of ROS generation [62].

Lactobacillus strains such as *Lactobacillus casei* BL23 and *L. acidophilus* LA5 may also protect from oxidative damage through mechanisms such as the production of antioxidant enzymes (SOD and CAT) that dismutate free radicals to O_2_ and H_2_O_2_. LA5 also acts through the downregulation of the expression of cyclo-oxygenase 2 (COX-2), which is highly associated with ROS production and inflammation [63]. Other strains such as *L. johnsonii* BS15 have been demonstrated to improve the intestinal *Firmicutes/Bacteroidetes* ratio, indicating that the modulation of the gut microbiota could have the capability to improve the host redox state [64].

Different signaling pathways associated with the antioxidant mechanisms of probiotics in the host, including protein kinase C (PKC), nuclear factor erythroid-2-related factor 2 (Nrf2), silent information regulator 1 (SIRT1), and mitogen-activated protein kinase (MAPK), have been proposed. However, information regarding the correlation between the action of a single strain and its specific pathway is still lacking and requires further investigation [61].

In a meta-analysis of five RCTs that included women with gestational diabetes, probiotic supplementation led to a significant reduction in CRP (*p* < 0.0001), IL-6 (*p* = 0.0005), and malondialdehyde (MDA) (*p* < 0.00001), and an increase in NO (*p* = 0.003) and total antioxidant capacity (TAC) (*p* = 0.01) [65]. Similar results were obtained in another meta-analysis involving nine RCTs in which the TAC and glutathione (GSH) were significantly improved (*p* = 0.005 and *p* = 0.006, respectively, compared with placebo) after probiotic ingestion. Moreover, a reduction in plasma concentrations of the MDA (*p* = 0.05) was also detected [66]. Serum levels of IL-6, IL-10, TNF-α, and MDA were also improved in patients with bipolar disorder treated for 8 weeks with a specific mix of lactic probiotics [67].

In a preliminary study, two specific strains of *Lactobacilli* (1:1 *L. rhamnosus* IMC 501 and *L. paracasei* IMC 502; 10^9^ cells/day) were supplemented for 4 weeks to evaluate the effect of probiotics on oxidative stress and their potential role in the neutralization of ROS in athletes during a 4-week period of heavy physical activity. At the end of the study, while the *Lactobacillus* count remained almost the same in the control group (5.2 ± 0.5 Log_10_ CFU/g of feces), in the group treated with probiotics, it significantly increased (*p* < 0.05), with a value of 6.6 ± 0.8 Log_10_ CFU/g of feces. In addition, a significant effect of probiotic supplementation on biological antioxidant potential was highlighted in comparison with the control group (*p* < 0.01). The potential levels of biological antioxidants after exercise were significantly higher in the active group than in the control group (*p* < 0.01). Moreover, the interaction between treatment and exercise on the potential levels of biological antioxidants was also significant (*p* < 0.01), confirming that probiotic supplementation significantly increased plasma antioxidant levels [68].

The combination of *Lactobacillus rhamnosus* IMC 501 and *Lactobacillus paracasei* IMC 502 was also investigated in a double-blind placebo probiotic feeding study (25 fed probiotics and 25 fed placebo) involving 50 healthy volunteers and a duration of 12 weeks. At the end of the intervention, a significant increase in fecal *Lactobacilli* and *Bifidobacteria* was observed in the probiotic group, and stool frequency and stool volume were higher in the probiotic group than in the placebo group [69].

In another trial, 36 subjects were divided to receive Vigiis 101-LAB (capsules produced from the fermentation of *Lactobacillus paracasei* subsp. paracasei NTU 101, 5 billion CFU/day) or placebo, for 4 weeks. At the end of treatment, *Bifidobacterium* spp. and *Lactobacillus* spp. counts were significantly higher in the feces of treatment subjects, with 4.01- and 4.25-fold increases, respectively. In addition, the same supplementation (10 billion CFU/day) in 52 subjects was found to have improved motility, decreased food transit time, and significantly increased immunoglobulin (Ig) G, IgM, and antioxidant activity [70].

A recent meta-analysis of a total of 26 RCTs (*n* = 1891) indicated that probiotics significantly improved gut barrier function measured by the levels of transepithelial resistance (*p* < 0.00001), serum zonulin (*p* = 0.0007), endotoxin (*p* = 0.005), and LPS (*p* = 0.02). Furthermore, probiotic groups were confirmed to have efficacy in reducing inflammatory factors, including CRP, TNF-α, and IL-6 [71].

In conclusion, the use of specific probiotic strains, taken regularly and in adequate dosages (at least 1 billion CFU/day), has been shown to improve the microbiota composition and therefore the intestinal homeostasis, reducing the production of ROS and inflammatory metabolites and the risk of endotoxemia. The prevention of gut aging and oxidative stress associated with the reduction in the risk of low-grade systemic inflammation by using probiotics appears to be the basis of the protection of different intestinal and extra-intestinal diseases. However, several aspects should be considered and further investigated in order to reduce their significant influence on the final effectiveness of the treatments: the choice of probiotic strains, the duration of supplementation, correct vehicles, and the timing of administration. Moreover, further randomized preclinical and clinical trials are required to confirm the preliminary evidence and better understand the mechanisms related to probiotic administration and the prevention of ox-inflammaging.

### 3.2. Probiotics and Urinary Tract Inflammation and Infections

Urinary tract infections (UTIs) are the most common bacterial infections worldwide after otitis media [72]. More than 150 million subjects/year worldwide suffer from UTIs, with an economic burden of > USD 2.6 billion in annual healthcare expenditures [73].

UTIs can be defined as pyelonephritis and kidney infections when affecting the upper urinary tract (kidney parenchyma and ureters) and cystitis and urethritis when affecting the lower urinary tract (urethra or bladder). Generally, UTIs are divided into sporadic, uncomplicated, and complicated infections [74].

Uncomplicated UTIs are highly prevalent in women, who, during their life, have a 50% risk of experiencing at least one episode of cystitis (vs. 12% risk in men) and a 20–30% risk of recurrent UTIs [75]. In this regard, conventional therapy, which is based on the use of antibiotics, represents the common approach to UTIs, despite presenting some weaknesses such as the risk of antibiotic resistance and damage to the gut microbiota [76]. In fact, the treatment of recurrent UTIs includes multiple antibiotics for different cycles during the year, also used as prophylactic agents. In this context, the rate of fluoroquinolone resistance is >20% in several nations, and in 2016, the FDA highlighted how fluoroquinolone-associated side effects generally outweigh its benefits for people with uncomplicated UTIs [77]. Recurrences in UTIs could be caused by the same or a different microorganism. *E. coli* is responsible for 85% of cystitis even if some Gram-positive bacteria such as *Staphylococcus saprophyticus*, some Gram-negative bacteria such as *Klebsiella pneumoniae*, and some enterococcal species may be involved in the pathogenesis of uncomplicated UTIs, directly adhering to the bladder epithelium [78]. The fecal–perineal urethral route, known as the ascending retrograde route, is recognized as the main route of infection, consisting of the colonization of the vaginal introitus and/or the urethral meatus by fecal microbiota-derived bacteria, with the consequent colonization of the bladder through the urethra [79]. In this regard, the intestine may act as a reservoir of uropathogens (“gut–bladder axis”), and thus, it plays an important role in UTI pathogenesis and the crosstalk between the intestinal and urogenital microbiome [80]. In addition, alterations in intestinal tight junctions in people with the “leaky gut syndrome” could represent another mechanism for visceral organ crosstalk, increasing the risk of bacterial material in the bladder, inflammation, and risk of UTIs [81]. In this context, probiotics can be used to alter bacterial colonization.

*Lactobacillus* species dominate the healthy vaginal microbiota and can inhibit the growth of several microorganisms, such as *Peptostreptococcus* spp., *Gardenerella vaginalis*, *Mobiluncus* spp., and *Bacteroides* spp. [82]. This action may be explained through different mechanisms of action, including the production of lactic acid, which reduces the vaginal pH; preventing the proliferation of nonindigenous organisms in the vagina [83]; and the defense of the genital mucosa through the creation of a biofilm that prevents the adhesion and growth of pathogenic microorganisms [84].

The probiotic combination of *Lactobacillus paracasei* IMC 502 and *Lactobacillus rhamnosus* IMC 501 has been shown to inhibit in vitro the adhesion of *Candida* species from vaginal cells and adhere to the vaginal epithelium [69]. In this context, in a preliminary study that included 35 healthy women, the supplementation with probiotic suppositories containing *Lactobacillus rhamnosus* IMC 501 and *Lactobacillus paracasei* IMC 502 (a matrix containing at least 10^9^ CFU of viable lactobacilli in a 1:1 combination) for 7 days contributed to a significant increase in the *Lactobacilli* level and restoring and maintaining a normal vaginal microbiota [85]. Even *L. gasseri* LG050 was shown to colonize the vaginal epithelium and restore the vaginal microbiota [86].

The probiotic supplementation per os of *L. reuteri* (former *L. fermentum*) and *L. rhamnosus* in premenopausal women with bacterial vaginosis was found to reduce vaginal coliforms and yeasts and improve the vaginal microbiota within four weeks of probiotic use [87]. In addition, the supplementation of *L. rhamnosus* GR-1 and *L. reuteri* RC-14 in premenopausal women with UTIs, along with antibiotic therapy, decreased UTI recurrence from 47% to 21% [88]. A similar study demonstrated a reduction in UTI recurrence from 6 to 1.6 per year using the probiotic combination *L. reuteri* RC-14/*L. rhamnosus* GR-1 [89]. An RCT of 252 postmenopausal women with recurrent UTIs, who were treated with the combination L. *rhamnosus* GR-1/*L. reuteri* RC-14 against daily prophylaxis with trimethoprim–sulfamethoxazole (480 mg/day), showed that individuals who received antibiotics reported an average of 2.9 UTIs in 12 months, whereas the active group averaged 3.3 UTIs, a result that did not meet the noninferiority criteria. However, an added benefit to taking the oral probiotic was a decreased level of antibiotic resistance [90].

A recent systematic review of a total of nine studies (772 adult patients) showed that probiotics may be a potential option to reduce UTIs [91]. In particular, *Lactobacillus crispatus*, *Lactobacillus acidophilus* PXN 35 [92], and *Lactobacillus plantarum* PXN 47 [93] reduced the risk of UTIs by nearly 50%.

Although the oral administration of different probiotic strains demonstrated potential efficacy in UTI prevention, no RCTs have yet quantitatively evaluated the effect of probiotics on the urinary microbiota. Therefore, it is unclear if probiotics, when administered orally, will colonize the adult female lower urinary tract and/or alter the existing urinary microbiota. Moreover, future studies should focus on more clinically relevant patient groups who are at higher risk of UTIs, considering possible differences with the vaginal administration and clarifying the duration of treatments, dosages, mode of administration, and eventual combinations with other nutraceuticals.

### 3.3. Probiotics and Cardiovascular Inflammation and Oxidative Stress

**Figure 3:**
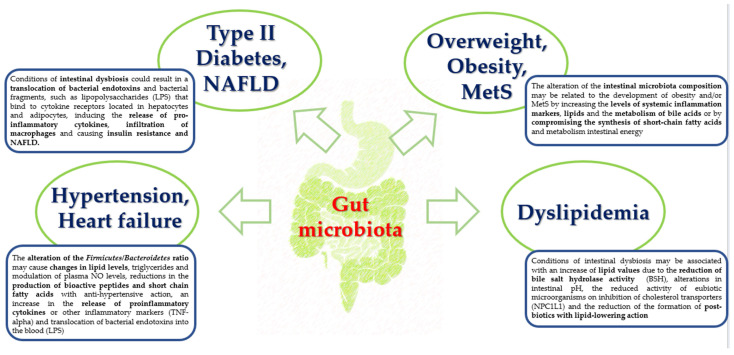
Alterations in the gut microbiota, inflammaging, and risk of being overweight, obesity, metabolic syndrome (MetS), dyslipidemia, type II diabetes, nonalcoholic fatty liver disease (NAFLD), hypertension, and heart failure.

The role of probiotics in reducing cardiovascular risk and oxidative stress is an aspect that has gathered scientific interest, especially considering new scientific evidence supporting the role of gut microbiota in the pathogenesis of CVDs [94]. Figure 3 highlights the significant role of the associated inflammaging of gut microbiota in the pathogenesis of different risk factors and CVDs (Figure 3). Currently, it is well established that a direct correlation exists between chronic low-grade systemic inflammation and oxidative stress caused by gut dysbiosis and the development of insulin resistance, diabetes, obesity, and other cardiovascular diseases [95]. The relative abundance of the four main phyla that populate the intestine, namely *Bacteroidetes*, *Firmicutes*, *Actinobacteria*, and *Proteobacteria*, may be associated with CVDs [96]. According to these considerations, in obese people, data confirmed that the *Firmicutes* phylum is generally more abundant than *Bacteroidetes*, whereas subjects with normal weight exhibited a considerable displacement toward the *Bacteroidetes* [97]. Another finding supporting this evaluation indicates that the weight loss induced by diet and/or bariatric surgery promotes significant changes in intestinal microbial composition [98]. The regulation of intestinal microbiota through the supplementation of probiotics was found to reduce low-grade intestinal inflammation and oxidative stress and improve the integrity of the intestinal barrier, promoting the homeostatic–metabolic balance and potentially reducing the risk of CVDs [99]. In this regard, the intestinal permeability may be altered following conditions of gut dysbiosis and therefore could cause the passage of opportunistic or pathogenic bacteria and/or bacterial fragments such as LPS through the intestine into the blood, leading to metabolic endotoxemia [100]. The LPS binds to cytokine receptors located in hepatocytes and adipocytes, thereby inducing the release of proinflammatory cytokines and causing insulin resistance. These molecules induce the infiltration of macrophages and play a role in the synthesis of inflammatory cytokines [101,102]. Probiotics can improve the functions of the intestinal barrier, thus promoting the proliferation of eubiotics or commensal microorganisms and inhibiting the proliferation of certain Gram-negative pathogens. They can also reduce the translocation of LPS and the production of proinflammatory cytokines in adipose tissues. The restoration of intestinal microflora after probiotic supplementation has been associated with an improvement in the functionality of the GLUT4 glucose transporter, the translation of PPAR-γ and lipogenic genes, and a reduction in the expression of inflammatory markers (IL-1β, IL-6, and TNF-α) [103].

Several studies have suggested that the gut microbiota could mediate low-grade inflammation classically associated with metabolic disorders related to obesity by exerting “anti-inflammatory” effects [104,105]. A decrease in IL-6 production, TNF-alpha, IL-1β and IL-6, and CRP was observed in different populations of both adult and elderly subjects [106]. Other probiotics may induce the production of anti-inflammatory cytokines.

A preliminary study that included 21 individuals with stable coronary artery disease showed that a 6-week daily supplementation with *Lactobacillus plantarum* 299v (Lp299v) had a favorable impact on the endothelium-dependent vasodilation of the brachial artery, through the increase in NO bioavailability, and it reduced the systemic inflammation, inducing changes in gut-microbiome-derived circulating metabolites [107]. Moreover, the administration of *L. helveticus* LBK-16H, for 21 weeks in 36 mildly hypertensive individuals reduced systolic blood pressure (SBP) on average by 6.7 (±3.0) mmHg compared with the control [108]. Similarly, a mean SBP reduction of 5.2 (±8.1) mmHg and diastolic blood pressure (DBP) of 1.7 mmHg was obtained in hypertensive men (aged 23 to 59 years) treated with the combination of *L. helveticus* and *S. cerevisiae* [109]. In another study, milk fermented with *L. casei Shirota* and *Lactococcus lactis*, enriched with GABA (1 mg/mL), showed a significant mean reduction in SBP (17.4 ± 4.3 mmHg) and DBP (7.5 ± 5.7 mmHg) in hypertensive patients [110]. In addition, a meta-analysis based on 14 RCTs showed that milk enriched with probiotics significantly reduced both SBP and DBP in grade I hypertensive patients [111]. In another RCT, the consumption of *L. plantarum* (2 × 10^10^/UFC/mL/day) in 36 smokers for 6 weeks significantly reduced SBP (13 ± 4 mmHg, *p* < 0.001). Furthermore, a significant reduction was observed in fibrinogen and cholesterol levels, leptin, IL-6, and F2-isoprostane concentrations (biochemical markers for lipid peroxidation and oxidative stress) [112].

A meta-analysis of several RCTs highlighted that when BP at baseline is high, greater anti-inflammatory and antihypertensive potential is achieved with at least two species of probiotics combined (especially *Lactobacilli* + *Saccharomyces*), a duration of treatment of ≥8 weeks, and a daily dose of ≥10^11^ CFU [113].

Other clinical and preclinical studies underline the key role of inflammaging of intestinal microbiota in heart failure; its prevalence is approximately 1–2% of the adult population in developed countries, which increases to 10% among people over 70 years [114]. Individuals with heart failure may manifest gastrointestinal disorders of absorption, motility, tissue perfusion, and edema, which cause alterations in the gut microbiota that, in the long term, are responsible for an increased risk of the translocation of endotoxins in the blood, causing an increase in the preload and afterload, and the aggravation of the clinical picture [115,116].

**Figure 4:**
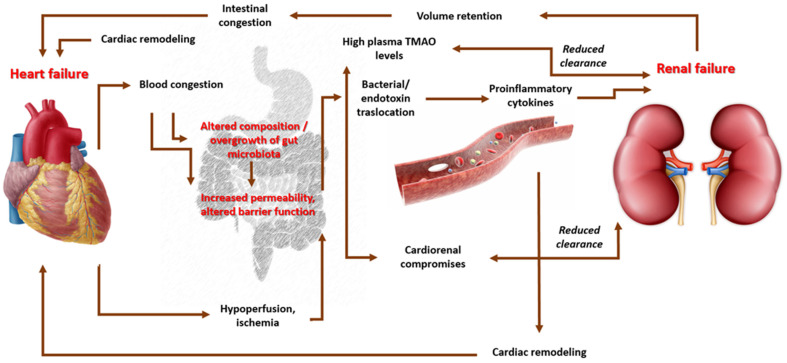
Correlations between intestinal dysbiosis and hemodynamic changes: modification in permeability and intestinal microbiota caused by associated leaky gut syndrome result in translocation in the bloodstream of microbes and endotoxins, followed by an increase in proinflammatory cytokine levels that could alter the renal clearance and cause heart failure. An altered gut microbiota is associated with an increase in trimethylamine N-oxide (TMAO) levels, which is also indirectly responsible for the exacerbation of heart failure and renal damage. The impaired clearance of these metabolites due to induced renal dysfunction further promotes the aggravation of the clinical picture by constituting a vicious circle.

In addition, a strong correlation exists between the severity of heart failure and the severity of intestinal dysbiosis, measured through the serum levels of trimethylamine-N-oxide (TMAO), an amine produced via the metabolism of choline and phosphatidylcholine from the intestinal microbiota. It is hypothesized that vascular remodeling and progressive coronary atherogenesis processes may be exacerbated in the context of high levels of TMAO [117,118]. The etiopathogenetic mechanism is not yet clear; however, it is evident that there is a direct proportional association between the blood levels of TMAO and the increase in intestinal edema, inflammatory metabolites, and cardiac and vascular remodeling (Figure 4) [119]. In a recent prospective study, the potential pathophysiological role of the gut microbiota in heart failure and its relationship with mortality from all causes was examined; in particular, in 720 subjects, followed up for a duration of 5 years, the role of TMAO was investigated through an analysis of fasting blood samples. The highest TMAO levels were reported in patients with heart failure (mean TMAO levels: 5.0 μM) compared with healthy individuals (mean TMAO levels: 3.5 μM, *p* < 0.001), with a risk of mortality increased by 3.4 fold [120]. Finally, it has been shown that elevated TMAO levels modify lipid metabolism through changes in the functionality of reverse cholesterol transport, sterol metabolism, and modification of the quality and quantity of bile acids [121,122]. A randomized clinical trial conducted on patients with HF NYHA class II or III and LVEF < 50%, treated for three months with a preparation containing 1000 mg/day of probiotics (*S. boulardii*), evaluated the efficacy of this supplementation on different hemodynamic parameters. At the end of the three months of treatment, the group treated with probiotics exhibited a significant reduction in uric acid levels (−1.08, *p* = 0.014 vs. placebo: −0.01, *p* = 0.930), total cholesterol (−7, 63, *p* = 0.010 vs. placebo: −2.02, *p* = 0.603), and hsCRP (−0.23, *p* = 0.116 vs. placebo: +0.44, *p* = 0.011); an improvement in LVEF (6.6, *p* = 0.005 vs. placebo: +4.2, *p* = 0.173); and a decrease in the left atrial diameter (−0.29, *p* = 0.044 vs. placebo: +0.2, *p* = 0.079) [123]. Preclinical data showed comparable results to those of *L. rhamnosus* [124].

## 4. Discussion

Microbiota-targeted treatments may be proposed as a possible preventive or therapeutic option for specific age-linked conditions and could potentially play a role in regulating the health concerns that accompany aging and its associated inflammatory processes [125]. In this regard, different studies on the relationship between illnesses and alterations in the gut microbiota highlighted the important role of the “second brain” in the association between human health and disorders such as CVDs [126,127] and specific autoimmune disorders including celiac disease [128].

Investigations on probiotic supplementation have shown interesting protective effects that might be used to develop potential remedies for a better quality of life for the aging population [46,129]. The international market of probiotics, which are available in different forms such as functional food and beverage, powder, capsule, and tablet, is growing year by year. In addition, probiotics have also been studied in combination with other dietary therapies, including fibers and nutraceutical compounds [130]. 

An expert panel of the European Society for Primary Care Gastroenterology stated that specific probiotics that have been tested should be considered for use in the regulation of different intestinal symptoms, favoring gut eubiosis [131].

Probiotics could modulate the gut microbiota, representing one of the key interventions for regulating the healthy gut microbiome and improving an imbalanced gut and inflammation. However, although the current evidence is promising, the complete mechanisms through which probiotics moderate the gut and inflammation are not entirely clear yet, particularly in aging people. In addition, data regarding the different effects of using a single strain or a more diverse cocktail of different strains of probiotics are still lacking, as well as data on hard outcomes and information on the period of treatments, dosages, and modes of administration [132].

To date, several questions are worth exploring by conducting specific preclinical and clinical trials. Firstly, an in-depth study is needed regarding the multiple actions of gut microbiota in eubiosis and the relative consequences of dysbiosis. Secondly, new studies of fecal transplantation in humans are necessary to verify whether the changes in the gut microbiota induced by probiotics could be responsible for the restoration of intestinal permeability and the low-grade inflammatory state, reducing oxidative stress and preventing the aging of the microbiota. Thirdly, it is necessary to study, in vitro and in vivo, the production of postbiotics by different probiotic strains, as well as their anti-inflammatory effects. These open questions could clarify the mechanisms involved in the protective effects of specific probiotics and thus its real potential in the prevention of inflammatory dysbiosis-associated diseases. 

An important aspect that may influence the inter-variability of the gut microbiota composition is the assumption of drugs. Most of the drugs prescribed in the world have been developed for oral use and are absorbed in the intestine [133]. Recent evidence has shown that approximately one-fourth of nonantibiotic drugs inhibit the growth of at least one strain of the physiological microorganism of the gut, potentially contributing to intestinal dysbiosis and gut inflammation. Among the cardiovascular drugs, calcium antagonists and different antiarrhythmics seem to reduce the relative abundance of gut microbiota. In addition, proton pump inhibitors and some antidiabetics are among the most noncardiovascular drugs consumed by patients. In this regard, the microbiota–drug interaction is particularly significant and prompts greater attention to possible alterations and ox-inflammaging of the microbiome [134].

## 5. Conclusions

The intestinal microbiota is a kaleidoscopic community of microorganisms that synergistically cooperate with the host in several metabolic activities, influencing intestinal inflammation and regulating the immune function and aging processes. Aging is a physiological process that exacerbates oxidative stress and inflammatory phenomena. In this regard, given the fact that the elderly population is set to increase, the role of the gut microbiota and the prevention of gut eubiosis should not be ignored, as this consortia of microorganisms have been closely linked to both the onset and prevention of several intestinal and extra-intestinal pathologies. Studies conducted to date highlight the possibility of restoring the microbiota composition using specific probiotics strains, reducing the progression of pathogens and native pathobionts, and preventing the risk of leaky gut syndrome, which is strictly correlated with extra-intestinal diseases such as urinary tract infections and cardiovascular pathologies. Although some clinical trials support the use of probiotics as promising preventive agents against ox-inflammaging, further in vitro and in vivo studies are needed to evaluate the efficacy of different strains, dosages, periods of treatments, and effects on hard outcomes, before validating a prescription in clinical practice. Finally, the use of 16S rRNA sequencing on fecal samples may allow for the study of the associations of factors affecting structural characteristics of gut microbiota and the potential probiotic interventions in restoring gut eubiosis.

## Figures and Tables

**Table 1:** Diseases related to dysbiosis.

**Digestive Disorders**
Acute microbial diarrhea Traveler’s diarrhea Antibiotic-therapy-associated diarrhea *Clostridium difficile*-associated diarrhea Irritable bowel syndrome (IBS) Inflammatory bowel disease (IBD) *Helicobacter pylori*-associated gastritis
**Extra-Intestinal Disorders**
**Gut–reproductive axis:** infertility, ovarian dysfunction, ovarian cancer, and postmenopausal osteoporosis; **Gut–kidney/bladder axis:** chronic and acute kidney disease, nephrolithiasis, nephropathy, urinary tract infections, and overactive/painful bladder; **Gut–Skin axis:** psoriasis, acne vulgaris, dermatitis, eczema, wrinkles, and aging; **Gut–liver axis:** nonalcoholic fatty liver disease, liver inflammation, and hepatocellular carcinoma; **Gut–heart axis:** heart failure, cardiovascular disease, atherosclerosis, thrombotic events, and hypertension; **Gut–muscle axis:** muscle impairment, frailty, and sarcopenia; **Gut–brain axis:** stress, anxiety, depression, schizophrenia, cognitive decline, autism, and neurodegenerative diseases; **Gut–lung axis:** chronic obstructive pulmonary disease; **Gut–pancreas axis:** diabetes and pancreas cell inflammation; **Gut–bone axis:** bone demineralization and osteoporosis.

## Data Availability

Not applicable.
